# Heterogeneity in the Association Between Pneumococcal Vaccination and the Risk of Severe Community-Acquired Pneumonia in Elderly Inpatients: A Causal Forest Analysis

**DOI:** 10.3390/vaccines14010090

**Published:** 2026-01-16

**Authors:** Yunhua Lan, Ziyi Xin, Zhuochen Lin, Jialing Li, Xin Xie, Ying Xiong, Dingmei Zhang

**Affiliations:** 1 Department of Epidemiology, School of Public Health, Sun Yat-sen University, Guangzhou 510080, China; yunhua-lan@outlook.com; 2 Department of Immunization, Guangdong Provincial Center for Disease Control and Prevention, Guangzhou 510317, China; galion88@163.com (J.L.); xiexintu@163.com (X.X.); 3 Department of Quality Management and Evaluation, The First Affiliated Hospital, Sun Yat-sen University, Guangzhou 510080, China; xinziyi3@mail.sysu.edu.cn (Z.X.); linzhch3@alumni.sysu.edu.cn (Z.L.)

**Keywords:** community-acquired pneumonia, pneumococcal vaccine, elderly, causal forest, heterogeneity

## Abstract

**Background:** Community-acquired pneumonia (CAP) is a leading cause of morbidity and mortality in the elderly. While pneumococcal vaccination is a core preventive measure, it remains unclear whether its association with severe CAP is uniform across all elderly subgroups. Our study aimed to evaluate the overall association of pneumococcal vaccination with the risk of severe CAP in hospitalized patients aged ≥ 65 years and to explore potential heterogeneity in this association using a causal forest model. **Methods:** We conducted a retrospective cohort study of patients discharged between January 2023 and June 2025, aged ≥ 65 years, with a primary diagnosis of CAP. We used multivariable logistic regression to estimate the average association and a causal forest model to explore heterogeneous patterns in the conditional average treatment effect (CATE). **Results:** Among 1906 included patients (severe CAP: 924; non-severe CAP: 982), PPSV23 vaccination was independently associated with reduced odds of all-cause severe CAP (adjusted OR = 0.610, 95% CI: 0.401–0.930). The causal forest model yielded an average treatment effect (ATE) estimate of −0.112 (95% CI: −0.200 to −0.023), corresponding to an 11.2 percentage-point reduction in absolute risk. Exploratory analysis suggested potential heterogeneity: the association appeared most pronounced in patients aged 65–74 years (CATE = −0.122) and showed an attenuating trend in older groups. Age was the primary variable associated with heterogeneity, followed by hypertension, SARS-CoV-2 infection, and sex. **Conclusions:** In this observational cohort study, PPSV23 vaccination was associated with a reduced risk of severe CAP in elderly inpatients under strong assumptions of no unmeasured confounding. Exploratory analyses suggested potential heterogeneity in this association, which appeared to attenuate with advancing age and may be influenced by comorbidities. These hypothesis-generating findings indicate that further investigation is needed to determine whether prevention strategies should be tailored for the very old and those with specific chronic conditions.

## 1. Introduction

Community-acquired pneumonia (CAP) represents a significant infectious disease burden among the elderly population, being a major cause of hospitalization and mortality. In China, the in-hospital mortality rate for CAP ranges from 0.8% to 2.1%, exceeding 2.49% for severe cases [1], posing a substantial public health challenge. As global demographics shift towards an aging population, the societal impact of CAP is expected to intensify, straining healthcare systems worldwide [2,3]. Streptococcus pneumoniae is a common opportunistic pathogen. A meta-analysis by Luo et al. [4] reported its detection rate in elderly CAP patients to be between 12.3% and 17.8%. Consistent with this, Guo et al. [5] identified it as a primary pathogen in elderly CAP patients or those with comorbidities such as cardiovascular disease, renal failure, and diabetes. Moreover, the development of resistance to multiple antibiotics makes the treatment of pneumococcal diseases particularly challenging [6,7]. Vaccination, including both polysaccharide and conjugate vaccines, remains a cornerstone of prevention. Consequently, many national guidelines recommend pneumococcal vaccination for adults aged ≥ 65 years [2,8,9,10]. However, reported vaccine effectiveness (VE) in observational studies varies considerably [11,12,13], potentially due to heterogeneity in study populations.

Traditional regression models estimate average population-level effects but are limited in their ability to uncover heterogeneous treatment effects across individuals with differing characteristics. The causal forest, a novel machine learning method for causal inference, can effectively estimate heterogeneous treatment effects and identify subgroups that may derive greater or lesser benefit from an intervention. This approach has been successfully applied in similar epidemiological contexts to uncover heterogeneous treatment effects [14,15]. Our study leverages a clinical database, combining traditional logistic regression with the causal forest algorithm, to assess the overall association of pneumococcal vaccination with severe CAP in elderly inpatients and to explore potential heterogeneity of this association across age, comorbidity burden, and other clinical characteristics. The exploratory findings aim to generate hypotheses to inform future research on pneumonia prevention strategies for the elderly, especially high-risk subgroups.

## 2. Materials and Methods

### 2.1. Study Design and Population

This retrospective cohort study included elderly patients with a primary diagnosis of pneumonia who were discharged from the First Affiliated Hospital of Sun Yat-sen University (a 3000-bed tertiary referral centre, Guangzhou, China) between 1 January 2023 and 30 June 2025.

Inclusion criteria: (1) age ≥ 65 years; (2) primary diagnosis of pneumonia (ICD-10 codes: J12–J18); and (3) met the diagnostic criteria for CAP, defined as onset in the community-acquired context with the presence of pneumonia-related symptoms, signs, and radiological evidence, excluding tuberculosis, pulmonary tumors, non-infectious interstitial lung diseases, pulmonary edema, atelectasis, pulmonary embolism; and other related diseases.

Exclusion criteria: Patients with hospital-acquired pneumonia (HAP), defined as pneumonia that developed ≥ 48 h after hospital admission.

Patients were grouped according to the established diagnostic criteria for severe CAP [6], the severe group was defined as patients who met one major criterion or a specified combination of minor criteria (e.g., ≥3 minor criteria), while the remaining patients were categorized into the non-severe pneumonia group. Major criteria: (1) requirement for invasive mechanical ventilation; (2) development of septic shock requiring vasopressor therapy. Minor criteria: (1) respiratory rate ≥ 30 breaths per minute; (2) oxygenation index (PaO_2_/FiO_2_) ≤ 250 mmHg; (3) multilobar pulmonary infiltrates; (4) altered mental status/disorientation; (5) azotemia (blood urea nitrogen ≥ 20 mg/dL or ≥7.1 mmol/L); (6) hypotension (systolic blood pressure < 90 mmHg) requiring aggressive fluid resuscitation.

The Charlson Comorbidity Index (CCI) is a widely validated tool for assessing comorbidity, demonstrating high validity in predicting in-hospital mortality and adverse outcomes [16]. It provides a composite score encompassing multiple major chronic conditions, facilitating the adjustment of comorbidity burden—a key confounding factor—in multivariable models. Simultaneously, it can be incorporated as an effect modifier in the causal forest model to explore the potential impact of the overall comorbidity burden level on vaccine effectiveness. It also offers a supplementary measure of a patient’s overall frailty, contributing to a more comprehensive understanding of vaccine effectiveness heterogeneity across populations with varying health statuses. Therefore, our study employed the CCI to quantify the overall comorbidity burden of patients. The concurrent use of both the CCI and specific disease variables allows for a more nuanced characterization of comorbidity’s influence on vaccine effectiveness from the dual perspectives of “overall burden” and “specific conditions.”

### 2.2. Data Sources

Data were extracted from two primary sources:

First Page of Medical Records (FPMR) Database: Provided patient demographics (age, sex), clinical characteristics, comorbidities, hospitalization costs, clinical diagnosis and treatment information. Comorbidities were coded according to the ICD-10 classification and used to calculate the CCI.

Guangdong Provincial Vaccine Administration and Immunization System: This provincial immunization registry was linked to the FPMR database to obtain individual-level pneumococcal vaccination records. Vaccination status was defined as receipt of the 23-valent pneumococcal polysaccharide vaccine (PPSV23) prior to hospital admission.

Data linkage was performed using a unique patient identifier. All data were de-identified prior to analysis to protect patient privacy.

### 2.3. Definitions and Variables

The primary outcome was all-cause severe CAP. The exposure of interest was PPSV23 vaccination, defined as receipt of at least one dose of PPSV23 administered no less than 14 days prior to admission. The time since vaccination was also recorded for descriptive purposes.

Covariates included: (1) demographics, namely age and sex; (2) comorbidities, which comprised hypertension, SARS-CoV-2 infection status, and components of the CCI; and (3) clinical measures, including the CCI score and total hospitalization costs.

### 2.4. Statistical Analysis

#### 2.4.1. Descriptive and Multivariable Analyses

Continuous variables were compared between groups using Student’s t-tests or Mann–Whitney U-tests, as appropriate. Categorical variables were compared using chi-square tests or Fisher’s exact tests. Unadjusted odds ratios (ORs) with 95% confidence intervals were derived for severe CAP. Multivariable logistic regression was employed to estimate the adjusted odds ratio for the association between vaccination and severe CAP, controlling for age, sex, CCI, hypertension, and SARS-CoV-2 infection.

#### 2.4.2. Causal Forest for Heterogeneous Treatment Effects

We used a causal forest model implemented in the grf package in R to investigate potential heterogeneity in the association between vaccination and severe CAP. This machine learning approach estimates conditional average treatment effects (CATE) by constructing an ensemble of causal trees, each designed to partition the data to maximize heterogeneity in treatment effects.

Key features of our causal forest implementation:

Outcome: severe CAP (binary).

Treatment: PPSV23 vaccination (binary).

Covariates: age, sex, CCI, hypertension, SARS-CoV-2 infection, and individual comorbidities.

Model Tuning: Parameters including sample fraction, number of trees, and minimum node size were tuned via cross-validation to optimize performance.

Validation: We assessed model calibration using the test_calibration function, which evaluates the alignment between predicted and observed treatment effects.

Variable Importance: Measured by the frequency of covariate use in splitting across the forest.

The causal forest provides an estimate of the average treatment effect (ATE) and individual-level CATEs, allowing for exploratory assessment of potential subgroup differences.

All analyses were conducted in R 4.2.0 and SPSS 26.0, with statistical significance defined as *p* < 0.05.

## 3. Results

### 3.1. Baseline Characteristics

A total of 1906 patients met the inclusion criteria, comprising 924 in the severe CAP group and 982 in the non-severe CAP group. Univariate analysis (Table 1) revealed that patients in the severe group were significantly older (77.78 ± 8.19 vs. 76.13 ± 8.02 years, *p* < 0.001), had higher CCI scores [3 (IQR 2–4) vs. 2 (IQR 1–3), *p* < 0.001], a higher proportion of males (69.9% vs. 63.6%, *p* = 0.004), and incurred higher total hospitalization costs (90,700 CNY vs. 14,500 CNY, *p* < 0.001).

The prevalence of several comorbidities was significantly higher in the severe group, including congestive heart failure (34.7% vs. 11.7%), cerebrovascular disease (28.7% vs. 13.6%), moderate-to-severe liver disease (9.7% vs. 3.5%), diabetes without complications (34.0% vs. 27.5%), renal disease (18.7% vs. 11.0%), and hypertension (59.4% vs. 53.0%) (all *p* < 0.05).

A total of 103 patients had received PPSV23 prior to hospitalization. Vaccination-to-admission intervals ranged from 19 days to 14.13 years, with a median of 1.66 years, and the majority (85%) of vaccinated individuals having been inoculated within 5 years of admission. The vaccination rate was significantly lower in the severe group (4.1%) compared to the non-severe group (6.6%). The unadjusted odds ratio for the association between vaccination and severe CAP was 0.605 (95% CI: 0.401–0.912, *p* = 0.016). To assess the potential for a confounding effect of the “healthy vaccinee bias,” we compared the CCI scores between the vaccinated and non-vaccinated groups using the Mann–Whitney U test. The results showed no statistically significant difference in CCI scores between the two groups (Z = −1.384, *p* = 0.166). We did not conduct time-since-vaccination stratified analyses due to limited sample size, and thus cannot rule out waning immunity as a source of bias.

### 3.2. Multivariate Logistic Regression Analysis

After adjusting for age, sex, CCI, hypertension, and SARS-CoV-2 infection in a multivariable logistic regression model (Table 2), CCI (OR = 1.152, 95% CI: 1.108–1.197, *p* < 0.001), age (OR = 1.027, 95% CI: 1.016–1.039, *p* < 0.001), male sex (OR = 1.281, 95% CI: 1.052–1.560, *p* = 0.014), and hypertension (OR = 1.228, 95% CI: 1.018–1.481, *p* = 0.032) remained independent risk factors for severe CAP. PPSV23 vaccination remained significantly associated with reduced odds of severe CAP (OR = 0.610, 95% CI: 0.401–0.930, *p* = 0.021). A sensitivity analysis excluding hypertension yielded a nearly identical result (OR = 0.611, 95% CI: 0.401–0.930, *p* = 0.022), confirming the robustness of the finding.

### 3.3. Heterogeneity Analysis Using Causal Forest Model

#### 3.3.1. Overall Effect and Heterogeneity Test

**Figure 1:**
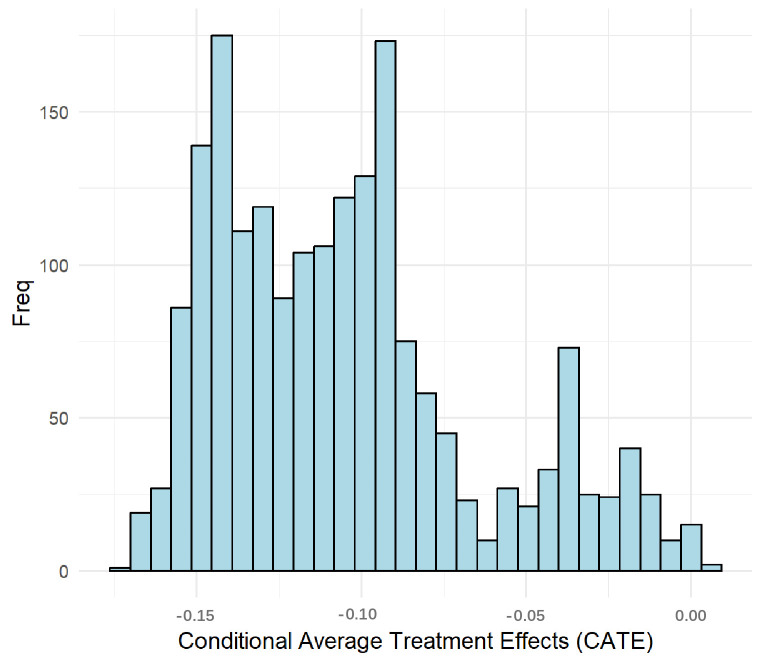
Distribution of Conditional Average Treatment Effects (CATE). The histogram shows the distribution of individual-level CATE estimates from the causal forest model. A negative CATE indicates a reduction in the risk of severe CAP due to vaccination.

The causal forest model estimated an ATE of −0.112 (95% CI: −0.200 to −0.023), indicating that vaccination was associated with an 11.2 percentage-point reduction in absolute risk of severe CAP. The distribution of CATE estimates was left-skewed (Figure 1), suggesting that most patients were estimated to derive some benefit from vaccination, with varying degrees of benefit. The Best Linear Fit test for the baseline outcome showed a coefficient of 0.985 (*p* = 0.009), close to the ideal value of 1, suggesting accurate prediction of baseline severe CAP risk.

However, the test for heterogeneity of treatment effects (HTE) was not statistically significant. This non-significant result, potentially attributable to sample size limitations or complex underlying heterogeneity patterns, is reflected in the treatment effect calibration coefficient of −0.018 (*p* = 0.506), indicating instability in the estimated individual-level CATE. To explore potential effect patterns of public health or clinical significance and generate hypotheses for future research, we subsequently conducted exploratory subgroup analyses.

#### 3.3.2. Variable Importance Analysis

**Figure 2:**
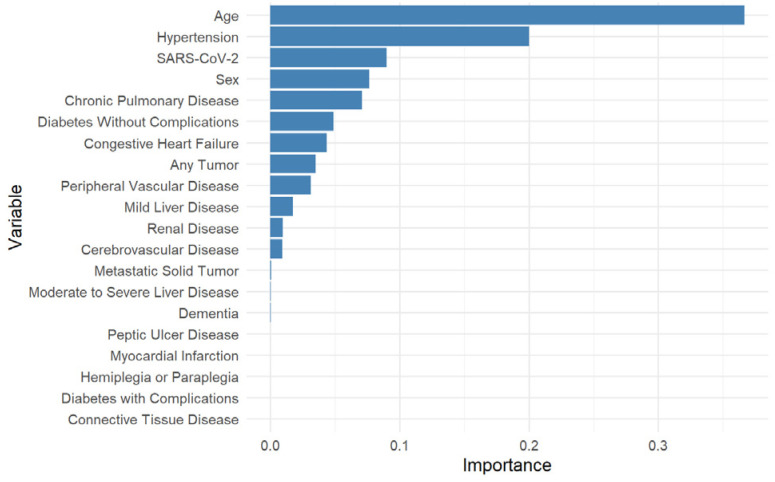
Variable Importance for Treatment Effect Heterogeneity. The bar plot displays the relative importance of each variable in modifying the association of pneumococcal vaccination, as estimated by the causal forest model.

In the exploratory variable importance analysis (Figure 2), age emerged as the top-ranking factor potentially modifying the vaccine’s association with the outcome, followed by hypertension, prior SARS-CoV-2 infection, sex, chronic pulmonary disease, and uncomplicated diabetes.

#### 3.3.3. Conditional Average Treatment Effects (CATE) in Key Subgroups

**Figure 3:**
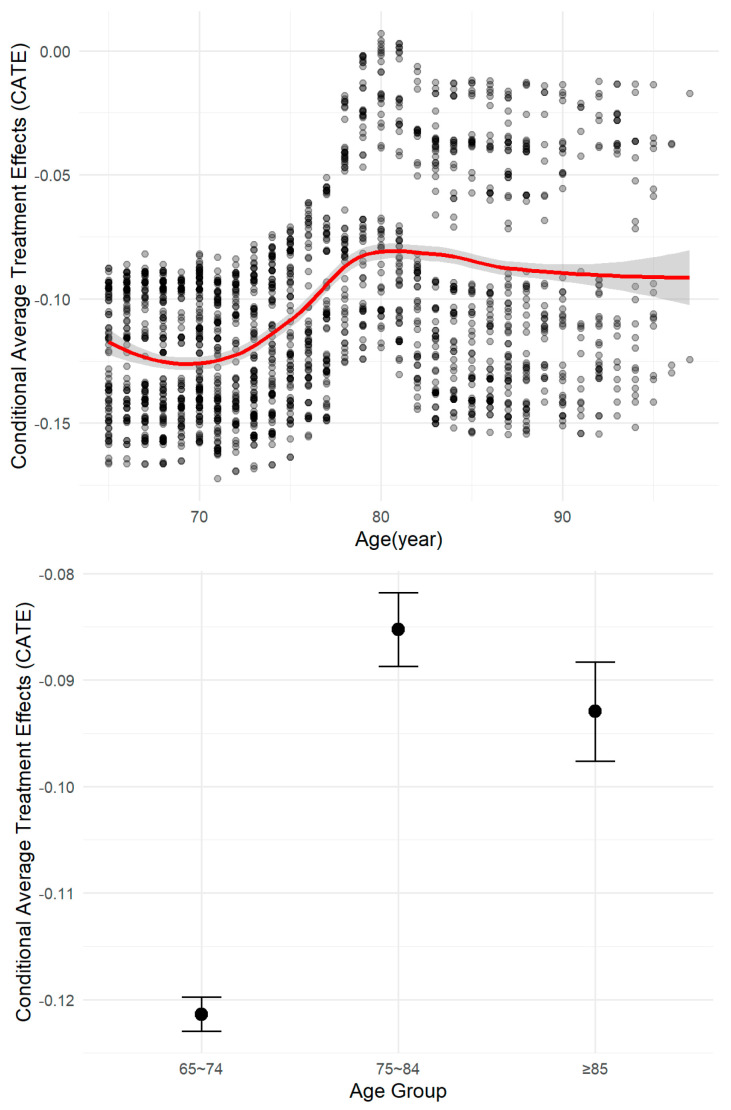
Conditional average treatment effect (CATE) by age. The boxplot distribution and mean CATE (central dot) for each group are shown. An exploratory smoothed line (red) with its 95% prediction interval (grey band) is overlaid to illustrate potential trends descriptively; these intervals are not for statistical inference.

In an exploratory analysis across age subgroups, a descriptive pattern was observed in the model-derived CATE estimates. The estimate was numerically largest in patients aged 65–74 years (CATE point estimate: −0.122), with numerically smaller estimates in those aged 75–84 years (−0.088) and ≥85 years (−0.093) (Figure 3).

**Figure 4:**
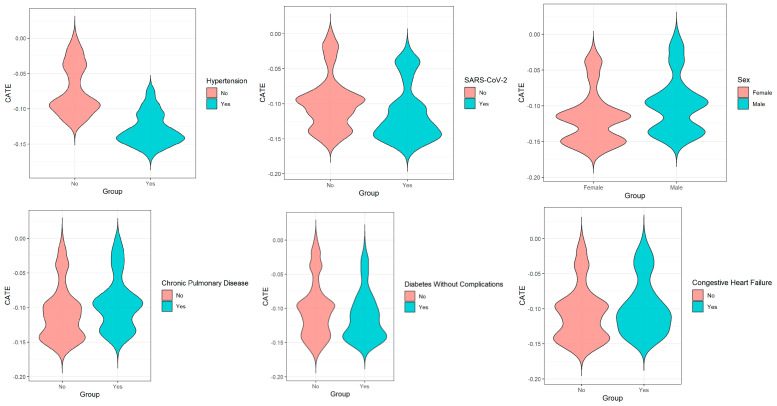
Exploratory Comparison of CATE Estimates Across Comorbidities Subgroup.

Exploratory subgroup analyses based on major comorbidities (Table 3 and Figure 4) showed numerical differences in the magnitude of association across patient groups. For instance, the point estimate was numerically stronger in patients with hypertension (CATE = −0.129) than those without (CATE = −0.074). Similarly, a numerically stronger association was observed among males (CATE = −0.117) and patients with SARS-CoV-2 infection (CATE = −0.114). Conversely, numerically weaker point estimates were observed in subgroups with conditions like renal disease (CATE = −0.115), chronic pulmonary disease (CATE = −0.108), or congestive heart failure (CATE = −0.107).

## 4. Discussion

This study applied a dual analytical approach—traditional multivariable regression and an exploratory machine learning method—to investigate the association between PPSV23 vaccination and all-cause severe CAP in elderly inpatients. Our principal findings indicate: (1) a significant overall association between PPSV23 vaccination and reduced odds of severe CAP; and (2) exploratory data-driven patterns suggesting potential variation in this association across patient subgroups, most notably by age. It is crucial to emphasize that the statistical test for heterogeneity of treatment effects (HTE) was not significant. Therefore, the subsequent discussion of subgroup trends should be interpreted strictly as hypothesis-generating, reflecting patterns of potential clinical interest that require formal validation in future studies.

The adjusted logistic regression analysis demonstrated a significant association between PPSV23 vaccination and a reduced risk of severe CAP (OR = 0.610). This finding aligns with a previous meta-analysis by Tin-Htar et al. [17], which reported that pneumococcal vaccines reduce the risk of severe pneumonia by 39–53% in the elderly. The causal forest model provided estimates consistent with this overall association (ATE = −0.112). The consistency of estimates from these distinct methodologies strengthens the evidence for an overall beneficial association within our study cohort. This reinforces current public health recommendations for pneumococcal vaccination in the elderly [2].

The most prominent pattern emerging from our exploratory analysis was a gradient across age subgroups. The point estimate of the association was largest in patients aged 65–74 years and appeared attenuated in older groups, particularly those ≥75 years. While these descriptive differences are constrained by the non-significant HTE and wider confidence intervals in the oldest subgroup (likely due to smaller sample size), the observed trend is biologically plausible. It is consistent with the concept of immunosenescence [18,19,20], wherein age-related declines in innate and adaptive immune function can impair vaccine responsiveness [21,22]. The T-cell-independent nature of the polysaccharide vaccine PPSV23, which does not induce robust immunological memory, may render its effectiveness particularly susceptible to such age-related immunological decline [23,24]. This pattern, though preliminary, suggests that the absolute benefit of vaccination might vary across the elderly population, potentially being highest in the “younger-old” cohort.

The variable importance ranking and exploratory subgroup analyses highlighted other factors, such as hypertension and specific comorbidities, as potential contributors to variation in the association. For instance, the point estimate was stronger in patients with hypertension. This intriguing observation could be hypothetically linked to studies suggesting pneumococcal vaccination may offer ancillary protection against cardiovascular events [25], or to a higher baseline risk of severe infection in this group [26], but it requires direct confirmation. Conversely, numerically weaker point estimates were observed in subgroups with conditions like chronic pulmonary disease or congestive heart failure. Chronic inflammation and immune dysregulation associated with these states could theoretically dampen responses to polysaccharide antigens [27]. However, in the context of a non-significant global test for heterogeneity, these between-subgroup differences must be viewed as descriptive and exploratory. They do not constitute evidence for definitive effect modification but rather serve to identify priority variables for investigation in future, adequately powered studies.

Our application of the causal forest model underscores both the promise and the challenges of using machine learning for heterogeneous effect exploration in observational clinical data. The model prioritized age as the most prominent potential modifier, a result concordant with strong biological prior knowledge, thereby lending face validity to the approach. However, the non-significant calibration statistic highlights the uncertainty inherent in estimating precise individual-level treatment effects with limited sample size, a known methodological challenge [28,29,30]. This outcome cautions against the over-interpretation of individual CATEs while affirming the utility of the approach for generating ranked hypotheses regarding variable importance. Notwithstanding these methodological cautions, the consistent overall association retains public health relevance. From a public health perspective, this significant association—observed even within a context of low vaccination coverage—supports ongoing efforts to increase vaccine uptake in the elderly. In China, pneumococcal vaccination coverage among the elderly remains generally low, with previous reports from the study region indicating coverage below 15% [31]. Our findings suggest that increasing vaccine uptake, even modestly, could yield considerable population health benefits. Given the high medical costs associated with severe CAP (averaging CNY 90,700 in this study), such an increase would also be economically advantageous, helping to conserve critical healthcare resources. Additionally, the exploratory finding regarding age suggests that immunization efforts could potentially be particularly beneficial if initiated among adults approaching 65 years, though vaccination remains important for all older adults given their elevated risk.

## 5. Limitations

The interpretation of our findings must be considered in light of several important limitations, which apply particularly to the exploratory causal forest analysis aimed at estimating heterogeneous treatment effects. First, our study is based on observational data, and the causal forest model operates under the strong assumption of unconfoundedness (i.e., that all important confounders have been measured and adequately adjusted for). Despite adjusting for available clinical and demographic variables, residual confounding from unmeasured or imperfectly measured factors—such as functional status, detailed smoking history, intensity of healthcare-seeking behavior, and socioeconomic status—remains possible. This inherent limitation of non-randomized studies precludes definitive causal conclusions. Therefore, our estimates, including the ATE and CATEs, should be interpreted as associations under the hypothetical condition of perfect confounding control rather than as proven causal effects. Second, the single-center, retrospective design may limit the generalizability of our findings to other settings or populations. The data reflect the practice and patient mix of a large tertiary referral center in Guangzhou, China. Third, the relatively small number of vaccinated patients, particularly in extreme age or comorbidity subgroups, limits the precision of subgroup estimates and the statistical power to detect true heterogeneity. Fourth, the outcome was all-cause severe CAP; without pathogen confirmation, we cannot ascertain the pneumococcal-specific effectiveness of PPSV23. Furthermore, data on pneumococcal conjugate vaccine (PCV) receipt were unavailable, precluding an assessment of their potential effect or interaction with PPSV23. Finally, as highlighted above, the non-significant heterogeneity test and the model calibration results indicate substantial uncertainty in the estimated individual-level CATEs. Consequently, the identified subgroup patterns should be viewed strictly as preliminary and hypothesis-generating, not as evidence of confirmed effect modification.

## 6. Conclusions

In conclusion, this observational study, under the assumptions of no unmeasured confounding, observed an inverse association between PPSV23 vaccination and severe CAP in hospitalized elderly patients. Exploratory analyses suggested potential heterogeneity in the strength of this association, with data-driven patterns pointing to age as a potential key modifier. However, definitive statistical evidence for heterogeneity was not established. These findings highlight two important considerations for future work: first, the observed overall association reinforces the current public health rationale for promoting pneumococcal vaccination in the elderly; and second, the exploratory patterns warrant targeted prospective research to verify whether the magnitude of benefit legitimately varies across subgroups defined by age and comorbidities. Such research should incorporate larger samples, pathogen data, and immunological endpoints to move from hypothesis generation to confirmed evidence, ultimately informing more precise prevention strategies.

## Figures and Tables

**Table 1:** Baseline characteristics of patients and univariate analysis.

Patient Characteristics	Frequency (*n* = 1906)	Non-Severe Group	Severe Group	Test Statistic (t/Z/χ^2^)	*p*-Value	OR (95% CI)
		(*n* = 982)	(*n* = 924)			
Age (mean ± SD)	1906	76.13 ± 8.02	77.78 ± 8.19	4.46	<0.001	-
CCI [M (Q1, Q3)]	1906	2 (1, 3)	3 (2, 4)	8.92	<0.001	-
Total hospitalization costs [M (Q1, Q3), CNY]	1906	14,500 (8900, 24,200)	90,700 (41,000, 198,700)		<0.001	-
Sex, (*n*, %)				8.42	0.004	1.327 (1.096~1.607)
Female	635	357 (36.4)	278 (30.1)			
Male	1271	625 (63.6)	646 (69.9)			
Myocardial infarction (*n*, %)				21.17	<0.001	4.764 (2.295~9.891)
No	1858	973 (99.1)	885 (95.8)			
Yes	48	9 (0.9)	39 (4.2)			
Congestive heart failure (*n*, %)				143.11	<0.001	4.013 (3.166~5.087)
No	1470	867 (88.3)	603 (65.3)			
Yes	436	115 (11.7)	321 (34.7)			
Peripheral vascular disease (*n*, %)				2.63	0.105	0.845 (0.69~1.036)
No	1393	702 (71.5)	691 (74.8)			
Yes	513	280 (28.5)	233 (25.2)			
Cerebrovascular disease (*n*, %)				65.01	<0.001	2.545 (2.019~3.207)
No	1507	848 (86.4)	659 (71.3)			
Yes	399	134 (13.6)	265 (28.7)			
Dementia (*n*, %)				4.29	0.038	1.595 (1.022~2.49)
No	1822	948 (96.5)	874 (94.6)			
Yes	84	34 (3.5)	50 (5.4)			
Chronic pulmonary disease (*n*, %)				2.14	0.143	1.166 (0.949~1.432)
No	1417	744 (75.8)	673 (72.8)			
Yes	489	238 (24.2)	251 (27.2)			
Connective tissue disease (*n*, %)				3.15	0.076	1.577 (0.95~2.619)
No	1842	956 (97.4)	886 (95.9)			
Yes	64	26 (2.6)	38 (4.1)			
Peptic ulcer disease (*n*, %)				4.65	0.031	2.073 (1.054~4.076)
No	1868	969 (98.7)	899 (97.3)			
Yes	38	13 (1.3)	25 (2.7)			
Mild liver disease (*n*, %)				2.03	0.154	0.845 (0.671~1.065)
No	1551	787 (80.1)	764 (82.7)			
Yes	355	195 (19.9)	160(17.3)			
Moderate-to-severe liver disease (*n*, %)				30.88	<0.001	3.009 (2.006~4.513)
No	1782	948 (96.5)	834 (90.3)			
Yes	124	34 (3.5)	90 (9.7)			
Diabetes without chronic complications (*n*, %)				9.43	0.002	1.357 (1.117~1.65)
No	1322	712 (72.5)	610 (66)			
Yes	584	270 (27.5)	314 (34)			
Diabetes with chronic complications (*n*, %)				2.59	0.108	1.565 (0.903~2.714)
No	1852	960 (97.8)	892 (96.5)			
Yes	54	22 (2.2)	32 (3.5)			
Renal disease (*n*, %)				22.60	<0.001	1.864 (1.438~2.416)
No	1625	874 (89)	751 (81.3)			
Yes	281	108 (11)	173 (18.7)			
Any tumor (*n*, %)				1.63	0.202	0.866 (0.695~1.08)
No	1503	763 (77.7)	740 (80.1)			
Yes	403	219 (22.3)	184 (19.9)			
Metastatic solid tumor (*n*, %)				2.46	0.117	1.179 (0.959~1.449)
No	1419	746 (76)	673 (72.8)			
Yes	487	236 (24)	251 (27.2)			
Hemiplegia or paraplegia (*n*, %)				*	1	1.063 (0.214~5.28)
No	1900	979 (99.7)	921 (99.7)			
Yes	6	3 (0.3)	3 (0.3)			
HIV/AIDS (*n*, %)				/	/	/
No	1906	982 (100.00)	924 (100.00)			
Yes	0	0 (0)	0 (0)			
Hypertension (*n*, %)				8.07	0.004	1.301 (1.085~1.56)
No	837	462 (47)	375 (40.6)			
Yes	1069	520 (53)	549 (59.4)			
SARS-CoV-2 infected (*n*, %)				1.42	0.234	0.894 (0.744~1.075)
No	1162	586 (59.7)	576 (62.3)			
Yes	744	396 (40.3)	348 (37.7)			
PPSV23 Vaccination (*n*, %)				5.85	0.016	0.605 (0.401~0.912)
No	1803	917 (93.4)	886 (95.9)			
Yes	103	65 (6.6)	38 (4.1)			

* Fisher’s exact test was used for statistical analysis of this variable due to the small expected frequency in some cells.

**Table 2:** Results of multivariate logistic regression analysis.

Factors	β	SE	Wals	OR (95%CI)	*p*-Value
CCI	0.141	0.02	51.084	1.152 (1.108~1.197)	<0.001
Age	0.027	0.006	21.044	1.027 (1.016~1.039)	<0.001
Sex					
Female (Ref)				1	
Male	0.247	0.101	6.052	1.281 (1.052~1.56)	0.014
Hypertension					
No (Ref)				1	
Yes	0.205	0.096	4.601	1.228 (1.018~1.481)	0.032
Vaccination					
No (Ref)				1	
Yes	−0.494	0.215	5.293	0.61 (0.401~0.93)	0.021

**Table 3:** Conditional average treatment effects (CATE) in comorbidity subgroups.

Variable	Subgroup	CATE Estimate (Mean ± SD *)
Hypertension	No	−0.074 ± 0.035
	Yes	−0.129 ± 0.022
SARS-CoV-2 infected	No	−0.099 ± 0.038
	Yes	−0.114 ± 0.039
Sex	Male	−0.117 ± 0.036
	Female	−0.099 ± 0.040
Chronic pulmonary disease	No	−0.108 ± 0.039
	Yes	−0.094 ± 0.038
Diabetes without chronic complications	No	−0.102 ± 0.041
	Yes	−0.112 ± 0.036
Congestive heart failure	No	−0.107 ± 0.040
	Yes	−0.098 ± 0.039
Any tumor	No	−0.107 ± 0.040
	Yes	−0.098 ± 0.037
Renal disease	No	−0.103 ± 0.040
	Yes	−0.115 ± 0.035

* The “±SD” here denotes the standard deviation of the individual CATE estimates within the respective subgroup, as generated by the causal forest model. This reflects model-based variability and heterogeneity within the subgroup, not the sampling variability of an independently observed sample mean. These values are provided solely for descriptive context regarding the dispersion of model estimates.

## Data Availability

The data presented in this study are available on request from the corresponding author. The data are not publicly available due to privacy and ethical restrictions.
